# Development of a multipurpose diagnostic marker using PCR, real-time PCR, and LAMP assays for rapid detection of *Rhizoctonia solani* in rice plants and soil

**DOI:** 10.3389/fpls.2026.1724916

**Published:** 2026-05-21

**Authors:** Asmita Singh, Prashantha S. Tadasanahaller, Sapna Sharma, Gopala Krishnan Subbaiyan, Rashmi Aggarwal, V. Shanmugam, Mahender S. Saharan, Bishnu Maya Bashyal

**Affiliations:** 1 Division of Plant Pathology, ICAR-Indian Agricultural Research Institute, New Delhi, India; 2 Division of Genetics, ICAR-Indian Agricultural Research Institute, New Delhi, India

**Keywords:** detection, disease management, LAMP, qPCR, *Rhizoctonia solani*, rice, sheath blight

## Abstract

*Rhizoctonia solani* AG1-IA is a major soil-borne pathogen responsible for sheath blight in rice, causing significant yield losses globally. Early, rapid, and accurate detection is essential for effective disease management, as traditional methods are time-consuming and less sensitive. In this study, we developed and validated a highly specific molecular marker, BrD3 targeting the bromodomain-containing Major Facilitator Superfamily (MFS) gene. This marker was successfully employed in PCR, quantitative PCR (qPCR), and Loop-mediated Isothermal Amplification (LAMP) assays for the detection of the pathogen in infected rice plants and soil samples. The PCR assay detected as low as 100 pg of DNA, while the qPCR assay demonstrated higher sensitivity, detecting 10 pg of DNA with high efficiency and accuracy (R^2^ > 0.99). Further, marker was utilized for the resistance evaluation against sheath blight disease of rice where highest copy no. of target gene was detected in susceptible rice variety Pusa Basmati 1121, and minimum copy no. was detected in resistant variety Tetep under inoculated conditions. The LAMP assay, optimized at 63°C with Hydroxy Naphthalol Blue (HNB) dye, provided rapid visual detection with a sensitivity of 1 ng DNA and no cross-reactivity with other rice pathogens. All three assays were validated in infected plants across multiple rice genotypes and soil samples of rice-wheat cropping system. The integrated diagnostic approach presented here offers a robust, specific, and field-deployable system for early detection and soil population monitoring of *R. solani* (AG1-IA) facilitating timely disease management.

## Introduction

Rice is an important food grain crop around the world. A soil-borne fungus *Rhizoctonia solani* causes sheath blight, root rot, and crown rot in a number of crops, including rice. *R. solani* can be divided into 14 anastomosis groups (AGs), namely, AG-1 to AG-13 and AG-BI (Carling et al., 2002). Sheath blight is one of the most devastating rice diseases in the world, which is caused by *R. solani* AG1-IA. There have been reports of sheath blight causing yield losses of roughly 50% worldwide depending on crop stage at time of infection, disease severity, and environmental conditions (Singh et al., 2004; Zheng et al., 2013; Bhunkal et al., 2015). Its significant genetic variability makes identification and detection more difficult (Gonzalez et al., 2006). *R. solani* is a necrotrophic fungus that produces sclerotia of varying sizes, which can remain dormant for many years (Mukherjee, 1978). Due to the pathogen’s longevity in soil and capacity to live in plant residues, conventional disease management techniques such as the application of chemical fungicides sometimes fall short of halting the spread of *R. solani*. Consequently, timely administration of control measures made possible by early and precise diagnosis of *R. solani*, which is important for disease management.

*R. solani* spreads through sclerotia present in soil and irrigation water (Senapati et al., 2022). Sheath blight is characterized by the development of lesions on the sheath, which may also spread to the leaves, higher sheaths, and even the panicle. The diseased sheath or leaves gradually dry out and die, which reduces the area of the canopy leaves and lowers output. Sometimes the disease spreads among other plants in the field or nursery and is not detected in time. Since the pathogen is soil-borne, it can endure for years in the soil. Therefore, early detection of pathogen in crop and soil is important. A quick and sensitive molecular diagnostic tool for managing sheath blight will be valuable in reducing the primary appearance of the disease, locating the pathogen in various host tissues and understanding the disease epidemiology.

Polymerase chain reaction (PCR) and DNA probes are frequently employed to identify pathogen causing plant diseases (Dubey et al., 2014; Sunani et al., 2019). PCR-based detection method has excellent efficiency, specificity, and sensitivity (Lin et al., 2009; Budge et al., 2009). Development of specific markers for detection has been recently used for number of pathogens, and this method greatly aids in distinguishing between various diseases (White et al., 1990; Robertson and Weller, 1998). Furthermore, a technology that has been widely used to identify plant pathogens is quantitative real-time polymerase chain reaction or qPCR (Lievens et al., 2006; Sayler and Yang, 2007; Lin et al., 2013). Sayler and Yang (2007) employed qPCR for the detection and measurement of *R. solani* AG1-IA. Assessing the level of host resistance or susceptibility, measuring the amount of inoculum in seed and soil for use in epidemiological investigations become easier with real-time PCR-based quantification (Aggarwal et al., 2017).

Loop-mediated isothermal amplification (LAMP), a visual detection method is a novel, innovative, unique gene amplification in which primer size and sequences are chosen in such a manner that Tm of primers fall between 60 °C and 65 °C so that amplification of the target gene can be completed in a simple water bath under isothermal conditions. There are few reports on development of LAMP assays for detection of *R. solani* and *R. zeae* causing soybean seedling blight and charcoal rot. Majority of these reports on specific detection of *R. solani* have employed the rDNA internal transcribed spacer (ITS) region (Ghosh et al., 2017; Lu et al., 2015).

Although several PCR, qPCR, and LAMP-based markers have been reported in the literature for the detection of *R. solani*, their availability remains limited and often restricted to specific genomic regions or assay conditions. However, no attempt was made to detect *R. solani* AG1-IA in soil and rice plant with multipurpose single primer targeting compatible across PCR, qPCR, and LAMP techniques. Therefore, the objective is to develop multipurpose highly specific and sensitive diagnostic assay to detect *R. solani* (AG1-IA) in soil and rice plant in real-world situations. In the present study, we developed bromodomain-containing (BrD) gene from the Major Facilitator Superfamily (MFS)-based molecular marker for PCR-, qPCR, and LAMP-based detection. Here, we developed a specific marker (110-bp product size) that was employed for both PCR- and qPCR-based detection of *R. solani* (AG1-IA) in soil samples and rice plants and the same genomic region was used to develop LAMP assay.

## Material and methods

### Sample collection

Previously characterized *Rhizoctonia solani* (**Supplementary Table 1**) isolates were used for most of the studies (Singh et al., 2023; Prashantha et al., 2021). Additionally, soil samples were collected from the infected rice plant rhizosphere and rice field practicing rice-wheat cropping system at ICAR-Indian Agricultural Research Institute, New Delhi, with map coordinates 28.6377° N, 77.1571° E. Field for the bakanae pathogen survival study (Sharma et al., 2024) was also utilized for the quantification of *Rhizoctonia solani* population. Samples were brought to the lab for DNA extraction and further molecular analysis.

### Isolation of DNA from *Rhizoctonia solani* and other fungal pathogens

Murray and Thompson (1980) cetyl trimethyl ammonium bromide (CTAB) technique was used to extract the genomic DNA. Isolates of *Rhizoctonia solani* obtained from rice host (TP3, TP10, and TP18)) along with different hosts which included turmeric (TP33), mustard (TP34), ginger (TP30), finger millet (TP31), foxtail millet (TP32), pakchoi (TP36), and two isolates of each of *Ustilaginoidea virens* (Uv2_4G and Uv 403), *Fusarium fujikuroi* (F32 and F55), *Bipolaris oryzae* (Bo1 and Bo4), and *Bipolaris sorokiniana* (Bs112 and Bs69 and Bs75) (**Supplementary Table 1**) were used. Approximately 220 mg of fungal mycelium was homogenized in precooled mortar and pestle with liquid nitrogen, and fine powder was obtained and transferred to 2-ml microcentrifuge tubes and 1 ml of CTAB buffer was added. Samples were then incubated in water bath at 60 °C–65°C for 1 h with gentle mixing at every 20 min by inverting the tube and then cooling down to room temperature. An equal volume of chloroform: isoamyl alcohol (24:1 v/v) was added and mixed by slight inversion. Samples were centrifuged at 10,000 rpm for 10 min at room temperature. Approximately 700 μl of supernatant was transferred (aqueous solution) to a clean sterile 2-ml microcentrifuge tubes. An equal volume (700 μl) of absolute ice-cold isopropanol was added and mixed by carefully inverting the tubes three to four times. The sample was again centrifuged at 10,000 rpm for 10 min, and supernatant was decanted. Obtained pellets were washed twice or thrice by adding 1 ml of 70% ethanol. Pellets were then air dried and 1× TE buffer was added and incubated at 37 °C for 1 h. To evaluate the purity of genomic DNA, the DNA was electrophoresed on a 0.8% agarose gel and quantified using a NanoDrop spectrophotometer (Thermo Fisher Scientific, USA).

### Extraction of DNA from plant samples

Rice genotypes were inoculated according to the method described by Bashyal et al. (2017). Briefly, the *Rhizoctonia solani*-colonized typha bits were tied in between the tillers just above the water level and control plants were inoculated with sterile non-colonized typha bits. Various sheath blight-infected genotypes of rice along with control sample (free of infection) were collected from the IARI field (MB4B), New Delhi, to validate the markers. Furthermore, a highly virulent *R. solani* isolate (TP18) was used to inoculate the rice varieties Swarna, Kali Khasa, Pusa Basmati 1, Rasi, and Pusa Basmati 1121 susceptible to sheath blight disease in order to test the marker effectiveness (Bashyal et al., 2017). At the symptomatic stage, tissue samples from diseased and healthy plants were collected and kept in a deep fridge (My Bio, USA). The CTAB method was used to extract genomic DNA from plant tissue (i.e., sheath) as described above.

### Extraction of DNA from soil samples

The pots, each containing 1 kg of field soil with natural organic matter, were inoculated with 3, 6, 9, 12, and 24 sclerotia in three replications under net house conditions, while maintaining adequate moisture to ensure sclerotial viability. After 7 days of inoculation, approximately 1 g of soil was collected from each pot. Furthermore, for quantification of the *R. solani* population in a rice–wheat cropping system field, soil samples were collected during different crop stages: July 2021 (rice crop), September 2021 (rice crop), December 2021 (wheat crop), and March 2022 (wheat crop) and kept in a deep fridge (My Bio, USA). Total DNA was extracted from all the soil samples using Zymo soil DNA extraction kit as per instruction manual provided.

### Identification of unique gene specific to AG1-IA for *Rhizoctonia solani* and primer designing

The comparative analysis was conducted in identifying species-specific proteins in *Rhizoctonia solani* anastomosis group AG1-IA using a comprehensive approach of proteome analysis and gene expression studies. To identify the genes involved in the interaction between *Rhizoctonia solani* and its rice host, a Pathogen Host Interaction (PHI) database-BLAST using the PHI-base (http://www.phi-base.org) was performed. A local BLAST search against the *Rhizoctonia solani* AG1-IA protein database identified putative pathogenicity-related genes (Prashantha et al., 2021).

Ultimately, the study identified a total of seven genes specific to AG1-IA: inorganic phosphate transporter (AG1_IPT), bromodomain containing protein (AG1_BrD), aldehyde dehydrogenase (AG1_AldD), AMP binding domain (AG1_AMP), and heme peroxidase (AG1_HmPr). Among these, AG1_IPT and AG1_BrD were found to be unique to *Rhizoctonia solani* AG1-IA. The bromodomain-containing protein (AG1_BrD) was then utilized for developing a diagnostic marker. Its protein sequence was converted into a nucleotide sequence, exhibiting 100% identity and query coverage with accession no. XM_043326099.1, which corresponds to the *Rhizoctonia solani* major facilitator superfamily transporter (RhiXN_06283) gene. Furthermore, primers were designed using the Oligo IDT Analyzer (Oligo Analyzer Tool - Primer analysis and Tm Calculator IDT) and examined for Tm, self-compatibility, hairpin structure, and cross primer binding (**Table 1**) and synthesized through Eurofins Scientific, New Delhi, India.

**Table 1:** Primers used in this study.

Pathogen	Name	Sequences (5'-3')	Tm of the primer
*Rhizoctonia solani*	BrD3 F/R	TGAACCTCTCCTTCTTTCCAAG*/*GATGCTGACGACATTACCATTG	58 °C
All fungal pathogens	ITS1/ITS4	TCCGTAGGTGAACCTGCGG/TCCTCCGCTTATTGATATGC	55°C
LAMP Primers	F3/B3	GCTTCGCCAGCAAGTTCT/ TTTCCGCTGAAGTTGGAGAC	56°C / 57.3°C
	FIP/BIP	TGTGCCAACAAAGAGGAGTGGTTTTTTCATTCACCCGCAGTTTGTCA/ACCCTCGTCAGGCAAGTTGAGTTTTTGGCTCGCTCGATTCGTCTA	73.8°C / 75.8° C

#### Optimization of PCR conditions

Purified genomic DNA of *Rhizoctonia solani* and other pathogens served as the template for PCR amplification. Sterilized ultrapure water (SUW) was included as a negative control. PCR reaction was carried out with 25–75 ng of genomic DNA, 2.5 μl of PCR 10× extraction buffer, 25 mM MgCl_2_, 2.5 mM each of dNTPs, 0.4 μM primer, and one unit of *Taq DNA polymerase* in the 25-μl reaction volume. The conditions of the thermal cycler (T100 Bio-Rad, USA) underwent initial denaturation at 95 °C for 3 min, 35 cycles of denaturation at 95 °C for 40 s, annealing at 56 °C for 40 s, and extension at 72 °C for 70 s. Final extension was operated at 72 °C for 7 min.

#### Specificity and sensitivity of the BrD3 marker

The specificity of the marker was tested by taking genomic DNA of the three distinct isolates of *R. solani* TP3, TP10, and TP18; two isolates each of *U. virens, F. fujikuroi*, *B. oryzae*, and *B. sorokiniana* were used for PCR amplification. Furthermore, the suitability of each DNA sample for the assays was confirmed by amplifying DNA from each isolate using the universal primers ITS1 and ITS4 (White et al., 1990). The specificity of the chosen primer pair was confirmed. A 1.4% (w/v) agarose gel containing ethidium bromide (0.5μg/ml) in 1× TAE buffer was used to visualize the PCR products. Furthermore, specificity of the marker was also tested in *R. solani* isolates taken from different hosts. To test the sensitivity of marker, different dilutions of *R. solani* genomic DNA were prepared which included 100 ng, 10 ng, 1 ng, 100 pg, 10 pg, and 1 pg, as described by Sunani et al. (2019). A visible fragment of the desired band in different dilutions served as an indicator of sensitivity.

#### Validation of *Rhizoctonia solani* specific marker

For validation of marker, genotypes of rice susceptible to sheath blight such as Swarna, Kalikhasa, Pusa Basmati 1, Pusa Basmati 1121, and resistant genotype Tetep were inoculated with highly virulent *Rhizoctonia solani* isolate TP18 under field conditions. At the symptomatic stage, diseased and healthy plant samples were collected and kept in a deep fridge (My Bio, USA). DNA was extracted from all the collected samples by the CTAB method, and the PCR amplification was performed with primer set BrD3 F/R. All PCR amplifications were performed in three independent experimental runs to ensure reproducibility of results.

### Real-time PCR assay

#### Optimization of the real-time PCR-based quantification

The same BrD3 primer, which was used in PCR was used in real-time PCR (qPCR) also. qPCR was optimized for parameters such as annealing temperature, primer concentration, and temperature to quantify the fluorescence signal of a particular amplicon. For all qPCR amplifications, SYBR Green fluorescent master mix dye (Thermo Scientific) was used. The following were the conditions of the LightCycler (Bio-Rad, USA): initial denaturation at 95 °C for 10 min followed by 40 cycles of PCR amplification at 94 °C for 15 s and 56 °C for 30 s, and default melt curve analysis. The optimized qPCR assay consisted of 2 μl (100 ng-10 pg) DNA, 1 μl of the forward and reverse primers each (10 μM), 10 μl of PCR master mix with SYBR Green buffer (Genetix, India) in a reaction volume of 20 μl. The volume was adjusted with nuclease-free water. All experiments were performed in triplicate. The negative control was double-distilled water. After 40 cycles, melting curve analysis (60 °C–95°C) was used to confirm the amplicons specificity (Sunani et al., 2019).

#### Development of standard curve

*Rhizoctonia solani* DNA was serially diluted in sterile ultrapure water (SUW) and in a fixed background of plant DNA isolated from rice sheath was utilized to create the DNA standard curve for the qPCR analysis. Standard calibration curve was obtained by plotting the Ct values against the logarithm of the genomic DNA concentration, followed by linear regression analysis to obtain the regression equation and correlation coefficient (R²). The experimental Ct values were converted into the amount of *Rhizoctonia solani* DNA (pg) in various samples using the standard regression lines as a reference curve. The amplification efficiency [E = 10 (−1/slope) −1] was calculated for the standard curve. Each dilution point used for standard curve construction was analyzed in triplicate, and the mean Ct value was calculated.

#### Application of qPCR assay for validation of diagnostic marker in resistant and susceptible rice genotypes

A group of susceptible genotypes, including Pusa Basmati 1121, Swarna, Kalikhasa, Rasi, and Pusa Basmati 1, were selected and inoculated with *R. solani* (isolate TP18)-colonized typha bits (Bashyal et al., 2017). Control plants (Swarna, Pusa Basmati 1121) were inoculated with sterile non-colonized typha bits. DNA was extracted after 5 days of inoculation following the CTAB method as mentioned above. One microliter of DNA isolated from plant tissue was used as a template for real-time PCR reactions in order to quantify the target DNA. By interpolating the cycle threshold (Ct values) of the sample DNA derived from qPCR with the standard curve, the concentration of the target DNA was determined. The number of copies of the target gene per gram of tissue was used to express the absolute amounts of *Rhizoctonia solani* total DNA. For real-time PCR analysis, three technical and three biological replicates were employed for each sample.

#### Quantification of *Rhizoctonia solani* population in soil samples under field conditions and in sclerotia-inoculated soil

The soil sample from the field was collected from different months, i.e., July 2021, September 2021, December 2021, and March 2022. For quantification of targeted DNA by qPCR, 1 μl of DNA extracted from soil was taken as template in real-time PCR reactions. The concentration of the target DNA was estimated by interpolating the cycle threshold (Ct values) of sample DNA obtained from qPCR with the standard curve. Total quantity of *R. solani* DNA was expressed as number of copies of the target gene.

### LAMP assay for detection of *Rhizoctonia solani*

#### Design of primers for LAMP detection assay

Four primers (F3, B3, FIP, and BIP) were designed from an identified specific sequence of major facilitator superfamily transporter (RhiXN_06283) gene using *Primer Explorer V4* software (http://primerexplorer.jp/e). LAMP primers used in the study are listed in **Table 1**.

#### Standardization of LAMP detection assay

The LAMP detection assay was performed in a 25-μl reaction volume. For standardization of protocol, concentrations of 25 mM MgSO_4_ (2 ± 4 μl) and 10 mM dNTPs (3 ± 4 μl) were taken. The concentrations of inner and outer primers to be used were also optimized. Betaine 2 μl (Sigma) and 1 μl of 8U *Bst polymerase* (New England Biologicals, USA) along with buffer was also added in the reaction mixture. The reaction mixture was preheated to 95 °C for 5 min and incubated at 63 °C-65 °C for 60 min in PCR (Bio-Rad, India). Furthermore, for termination of the reaction, the mixture was heated at 80 °C for 10 min. The turbidity of LAMP-amplified products were visually observed. Subsequently, 100 to 200 μM HNB dye was added to 10 μl LAMP-amplified products, to test the change in color reaction mixture. Finally, the banding pattern of LAMP products was noted by resolving on 1.5% (w/v) agarose gel.

#### Specificity and sensitivity test of LAMP assay

For determining the specificity of *R. solani* in LAMP assay, the same primer sets were used to amplify other pathogens *U. virens* (Uv2_4G and Uv403), *F. fujikuroi* (F32 and F55), *B. oryzae* (Bo1 and Bo4), and *B. sorokiniana* (Bs112 and Bs 69).

To test the sensitivity of the LAMP assay, total genomic DNA of *R. solani* was diluted to different concentrations, *viz.*, 100 ng, 10ng, 1 ng, 100 pg, 50 pg, 10 pg, 100 fg, and 10 fg and nuclease-free water was taken as non-template control (NTC).

#### Application of LAMP assay for validation of diagnostic marker in resistant and susceptible rice genotypes

To validate LAMP assay in plant samples, resistant and susceptible rice genotypes were inoculated with *R. solani* isolate as mentioned above. DNA was extracted from plant samples after 5 days of inoculation following the CTAB method as mentioned above. Furthermore, 2 µl of DNA isolated from plant tissue was used as a template for LAMP reactions in order to quantify the target DNA in 25-μl reaction volume and other reaction concentrations, as mentioned above.

## Results

**Figure 1:**
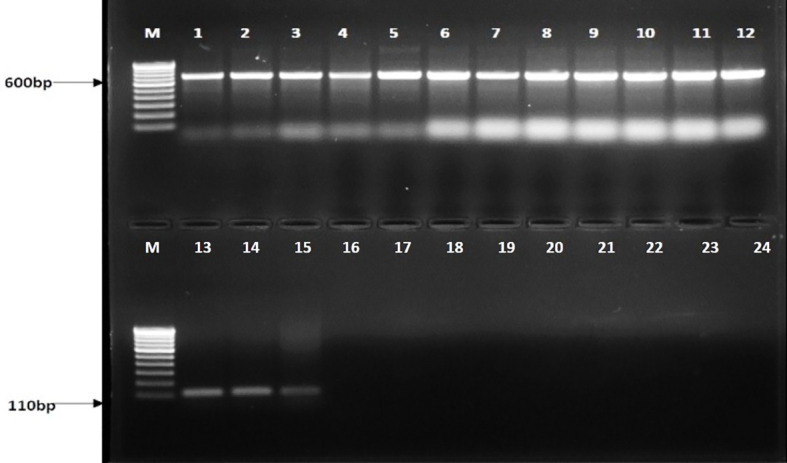
Agarose gel showing PCR amplification with different isolates taken for the study with ITS universal primers (ITS 1 and ITS 4) and BrD3F/R primers. M: 100-bp marker, lanes 1–3: *Rhizoctonia solani* isolate name (TP3, TP10, TP18) 4-5: *Ustilaginoidea virens (*Uv2_4G, Uv403*)*, 6-7: *Fusarium fujikuroi* (F32, F55), 8-9: *Bipolaris oryzae* (Bo1, Bo4), 10-12: *Bipolaris sorokiniana* (Bs112, Bs69 and Bs75), M: 100-bp marker, lanes 13-15: *Rhizoctonia solani* isolate name (TP3, TP10, TP18) 16-17: *Ustilaginoidea virens* (Uv2_4G, Uv403*)*, 18-19: *Fusarium fujikuroi* (F32, F55), 20-21: *Bipolaris oryzae* (Bo1, Bo4), 22-24: *Bipolaris sorokiniana* (Bs112, Bs69 and Bs75).

The primer set developed in this study was highly specific to *R. solani* AG1-IA isolates which produced a distinct 110-bp amplicon. This amplicon was absent in other pathogens such as *U. virens* (Uv2_4G and Uv403), *F. fujikuroi* (F32 and F55), *B. oryzae* (Bo1 and Bo4), and *B. sorokiniana* (Bs112 and Bs 69) and other host DNA samples taken for the study (**Figure 1**).

**Figure 2:**
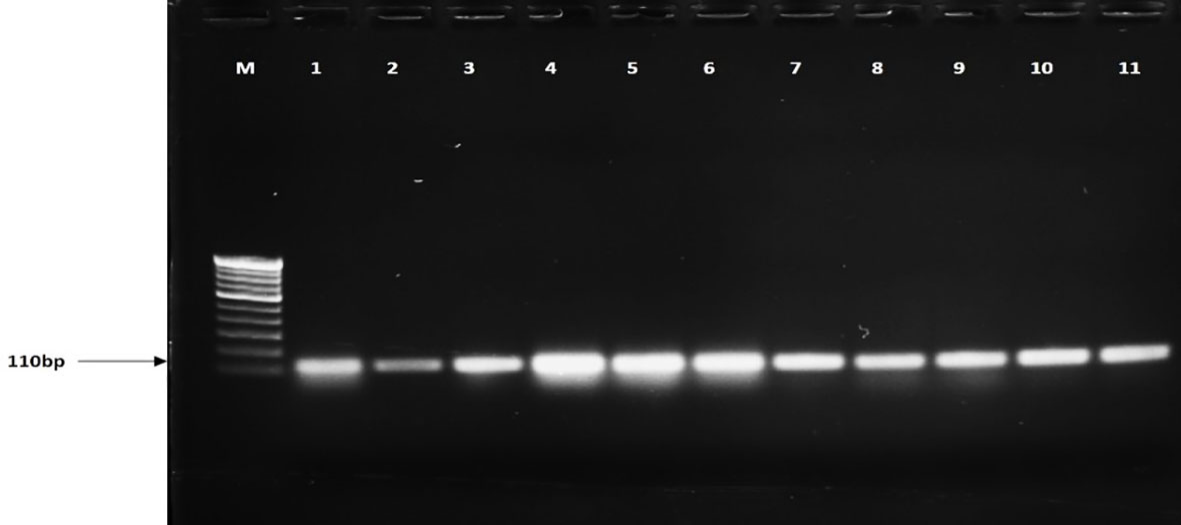
Detection of *Rhizoctonia solani* from different hosts. M: 100-bp marker, lanes 1-4: rice, 5: turmeric, 6: cabbage, 7: mustard, 8: ginger, 9: finger millet, 10: foxtail millet, 11: pakchoi.

**Figure 3:**
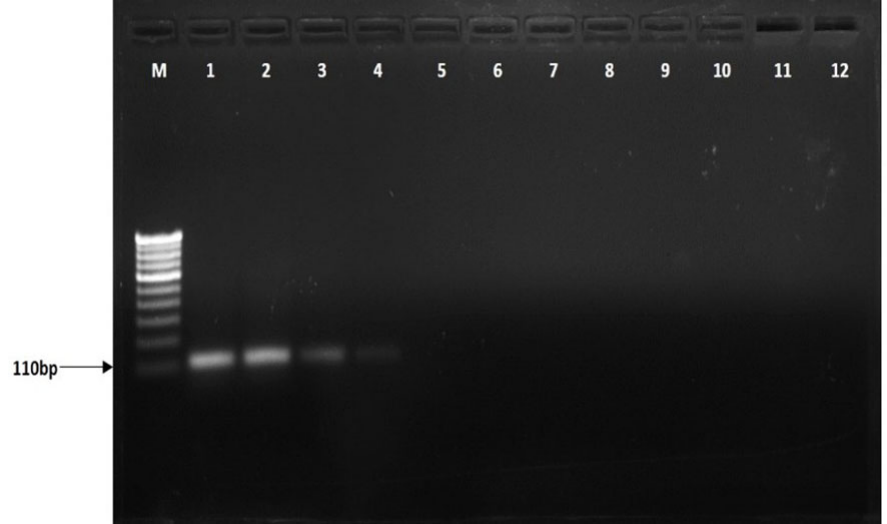
Sensitivity of BrD3 primers to detect different dilutions of *Rhizoctonia solani* DNA. M: 100-bp marker, lane 1:100 ng, 2:10 ng, 3:1 ng, 4:100 pg, 5:10 pg, 6:1 pg, 7:100 fg, 8:10 fg, 9:1fg 10: distilled water.

Additionally, the ITS region was amplified using ITS1 and ITS4 primers with the 600-bp fragment in all tested isolates, including *R. solani* and other pathogens (**Figure 1**). Furthermore, the BrD3 marker could amplify *R. solani* isolated from different hosts which included rice, turmeric, mustard, ginger, finger millet, foxtail millet, and pakchoi (**Figure 2**). The sensitivity of the marker was tested using a dilution series of total genomic DNA extracted from *R. solani* (TP3), which revealed that an up to 100-pg template was sufficient for conventional PCR-based detection assay (**Figure 3**).

### Detection of *Rhizoctonia solani* from field and soil sample using PCR

**Figure 4:**
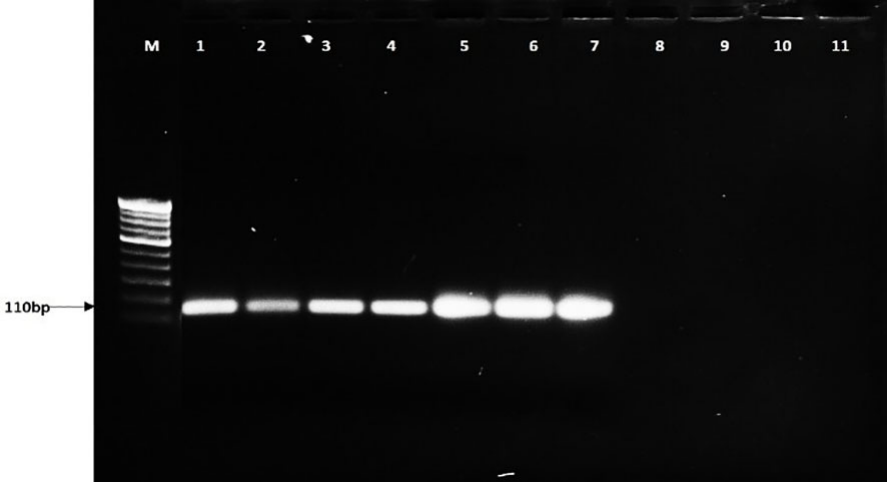
PCR-based detection of *Rhizoctonia solani* using BrD3 primers in different rice varieties. M: 100-bp marker, lanes 1-2: Pusa Basmati 1121, 3-4: Pusa Basmati 1, 5: Swarna, 6: Rasi, 7: Kalikhasa, 8: Tetep, 9: Pusa Basmati 1121 control, 10: Rasi control, 11: negative control.

**Figure 5:**
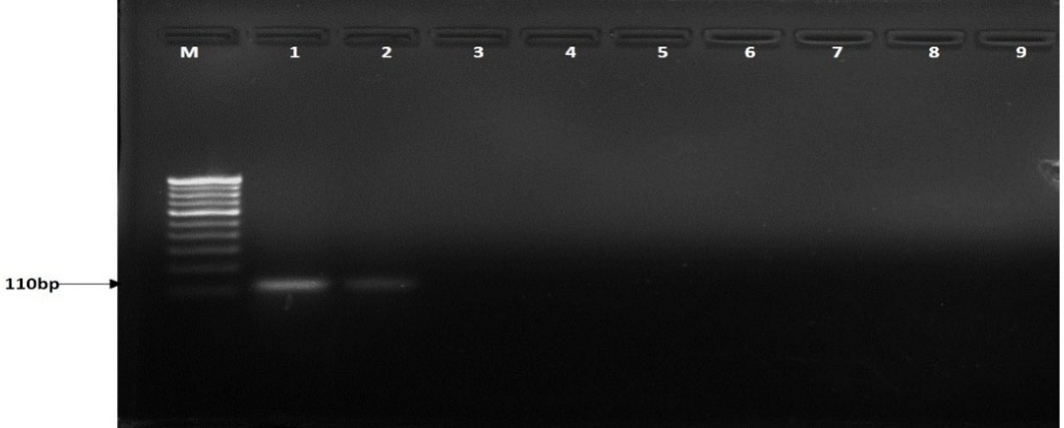
PCR-based detection of *Rhizoctonia solani* in field soil using BrD3 primers. M: 100-bp marker, lane 1: soil DNA sample from July 2021, 2: soil DNA from September 2021, 3: soil DNA from December 2021, 4: soil DNA from March 2022.

**Figure 6:**
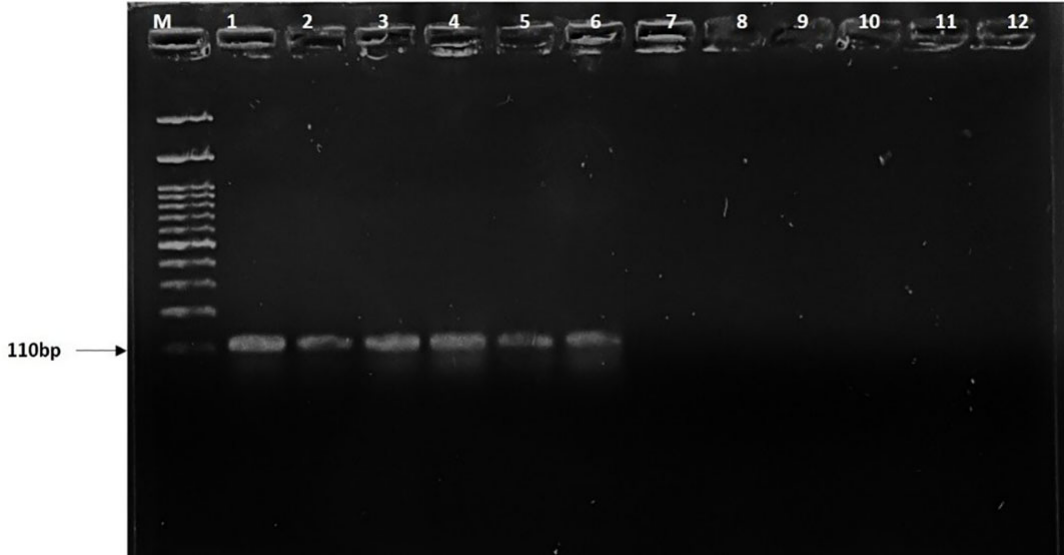
PCR-based detection of *Rhizoctonia solani* sclerotia in soil using BrD3 primers M: 100-bp marker, lanes 1-2: 24 sclerotia, 3-4: 12 sclerotia, 5: 9 sclerotia 6-7: 6 sclerotia, 8-9: 3 sclerotia, 10-11: control.

PCR-based detection assay showed a specific band of 110 bp in plant and soil samples inoculated with *R. solani*, whereas no band was observed in uninoculated and negative control samples. Amplification of the BrD3 region was observed in all the infected sheath samples of different rice varieties such as Pusa Basmati 1121, Rasi, Kalikhasa, Pusa Basmati 1, and Swarna. No amplification was observed in control plants (**Figure 4**). The marker could detect *R. solani* in soil samples of July and September 2021 (**Figure 5**). Soil samples inoculated with 9, 12, and 24 sclerotia showed the presence of *Rhizoctonia solani* with the band at 110 bp (**Figure 6**). Therefore, the marker developed in this study is proved to be effective for diagnosing *R. solani* from host plant and soil samples.

### Molecular detection of *Rhizoctonia solani* using qPCR

**Figure 7:**
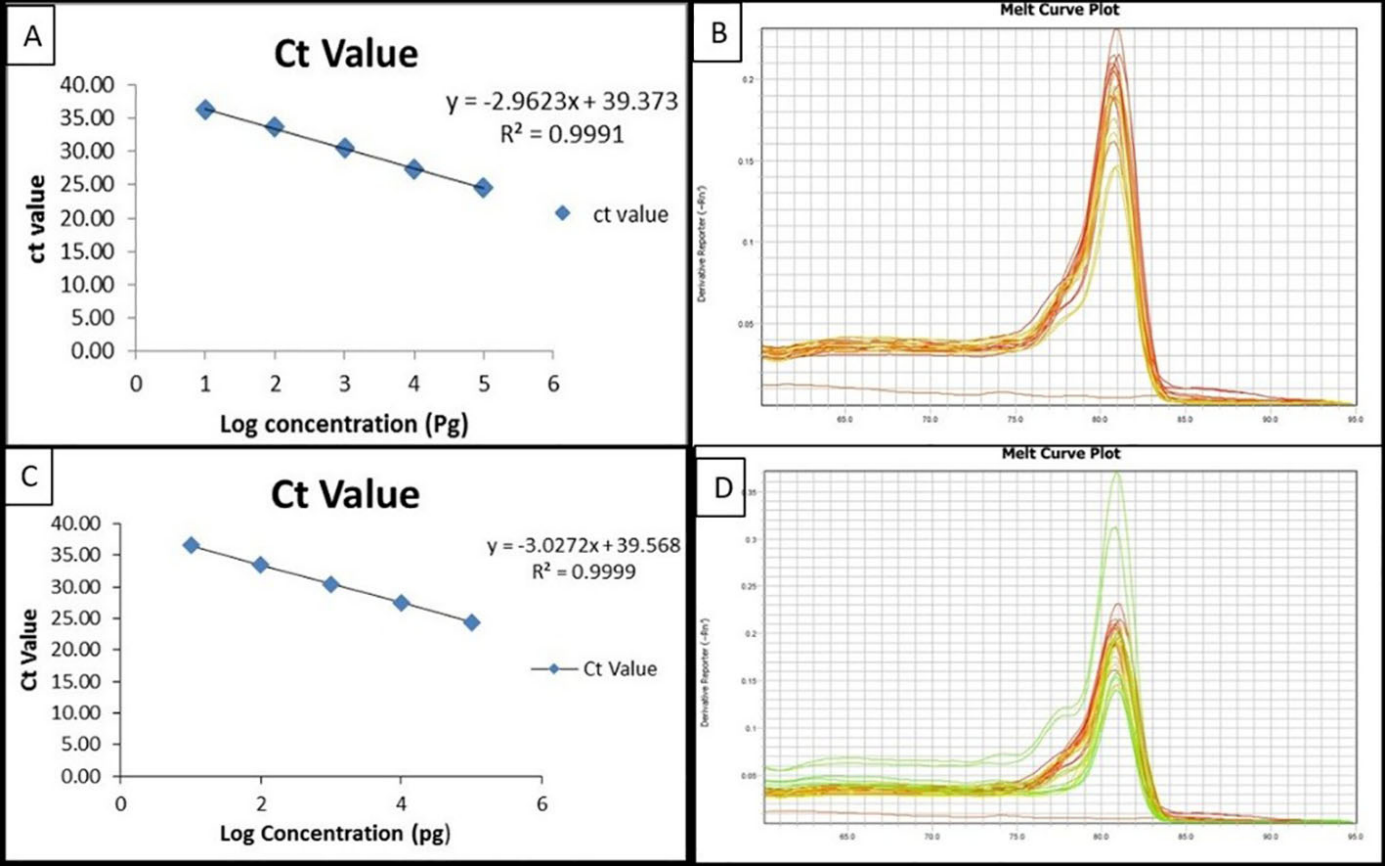
Standard curve and melting curve obtained through real-time PCR using *Rhizoctonia solani*-specific primers in plants and soil samples. **(A)** log_10_ DNA concentration plotted against the real-time PCR Ct, cycle threshold for different dilutions of pure plant DNA of *Rhizoctonia solani.* **(B)** Melting curve analysis through real-time PCR products using a *Rhizoctonia solani*-specific primer. **(C)** Standard curve showing the log_10_ DNA concentration plotted against the real-time PCR Ct, cycle threshold for different dilutions of pure genomic DNA of *Rhizoctonia solani* in soil samples inoculated with sclerotia. **(D)** Melting curve obtained through real-time PCR products using a *Rhizoctonia solani*-specific primer for the soil sample.

The BrD3 primer set was found effective and specific in PCR-based detection of *Rhizoctonia solani.* Therefore, it was further used in real-time-PCR based detection of *R. solani*. The standard curve drawn using *R. solani* DNA showed a linear correlation between Ct value and DNA concentration with a correlation coefficient of 0.9991 showing the accuracy and 117.58% efficiency, as indicated by the slope value (−2.962) of real-time PCR-based quantification (**Figure 7**). Target DNA showed fluorescence. Last fluorescent signals were observed at Ct 36.261 corresponding approximately to 10 pg DNA, whereas at Ct 24.575, it reached to the highest concentration of 100 ng. The sensitivity of the real-time PCR-based marker was found to be 0.1 ng corresponding to Ct value 33.655 with copy no.1934.167.

In the case of soil samples also, the standard curve showed a linear correlation between Ct value and DNA concentration with a correlation coefficient of 0.9999 and real-time efficiency of 114% as denoted by slope (−3.027) value (**Figure 7**). The marker was also used to monitor the soil population of *Rhizoctonia solani* in a rice-wheat cropping system, i.e., July 2021, September 2021, December 2021, and March 2022. Last fluorescence was observed at Ct 36.592 with DNA corresponding to approximately 10 pg. Specificity of the primers was confirmed by melting curve showing one distinct peak at 81 °C (**Figure 7**).

### Application of qPCR for validation of a diagnostic marker in resistant and susceptible rice genotypes

The qPCR-based marker BrD3 was used for the quantification of inoculums in different rice genotypes as mentioned above. Pusa Basmati 1121 was identified to be the most susceptible genotype with Ct value 27.345 (copy no. 261105), which was followed by Rasi with 30.195 (copy no. 28485), Swarna 30.988 (copy no. 15378), Kalikhasa 32.182 (copy no. 6078), and Tetep 33.379 (copy no. 2397). No amplification was observed in control plant samples. Samples showing Ct values of above 33 were considered negative for *R. solani* (**Table 2**; **Supplementary Figure 1**).

**Table 2:** Detection of *Rhizoctonia solani* in rice genotypes through different markers.

Rice genotype	Detection by PCR	Detection by real-time PCR	Copy number	Detection by LAMP
Pusa Basmati1121	+	+	261105	+
Rasi	+	+	28485	+
Swarna	+	+	15378	+
Kalikhasa	+	+	6078	+
Tetep	–	+	2397	_

### Application of qPCR for quantification of *Rhizoctonia solani* from soil

Real-time PCR was also used for the mapping of the *Rhizoctonia solani* soil population. Soil samples collected in July 2021 showed the highest inoculum (*R solani*) with a Ct value of 27.095 (copy no. 299468) followed by the September 2021 sample with a Ct value of 30.105 (copy no. 30341); the December 2021 sample showed a Ct value of 33.235 (copy no. 2806), whereas in March 2022, the Ct value was observed to be 34.885 (copy no.800) (**Table 3**).

**Table 3:** Detection of *Rhizoctonia solani* in soil samples through various assays.

Soil sample	Detection by PCR	Detection by real-time PCR	Copy number
Soil inoculated with 24 sclerotia	**+**	**+**	593817
Soil inoculated with 12 sclerotia	**+**	**+**	27069
Soil inoculated with 9 sclerotia	**+**	**+**	14180
Soil inoculated with 6 sclerotia	–	**+**	2150
Soil inoculated with 3 sclerotia	–	**+**	1541
Soil July 2021	**+**	**+**	299468
Soil, September 2021	**+**	**+**	30341
Soil, December 2021	–	**+**	2806
Soil, March 2022	–	**+**	800

Where, +, Positive; -, Negative.

In soil samples inoculated with 24 sclerotia, the Ct value observed was 26.195 (copy no. 593817) followed by 12 sclerotia with Ct value 30.555 (copy no. 27069), the sample inoculated with 9 sclerotia showed a Ct value of 31.105 (copy no. 14180), whereas the 6-sclerotia-inoculated sample showed Ct value 33.585 (copy no. 2150) and the soil inoculated with 3 sclerotia showed Ct value 34.023 (**Table 3**).

### Standardization of the LAMP detection assay

The optimized concentrations of different components in 25 μl reaction buffer are as follows: 2.5 μl of 10× thermo pol buffer, 1 μl of 8U *Bst polymerase*, 2 μl MgSO_4_ (25mM), 3.5 μl dNTPs (10mM), betaine 5μl (Sigma), 3 μl each of FIP and BIP, and 1.5 μl of each of F3 and B3 primers. The temperature gradient showed that the reaction mix incubated at 63 °C for 60 min was optimum for the assay developed in this study. Visual detection of LAMP reaction using 120 μM HNB dye was optimum for color change from violet to sky blue in positive reaction.

### Specificity and sensitivity of the LAMP assay

**Figure 8:**
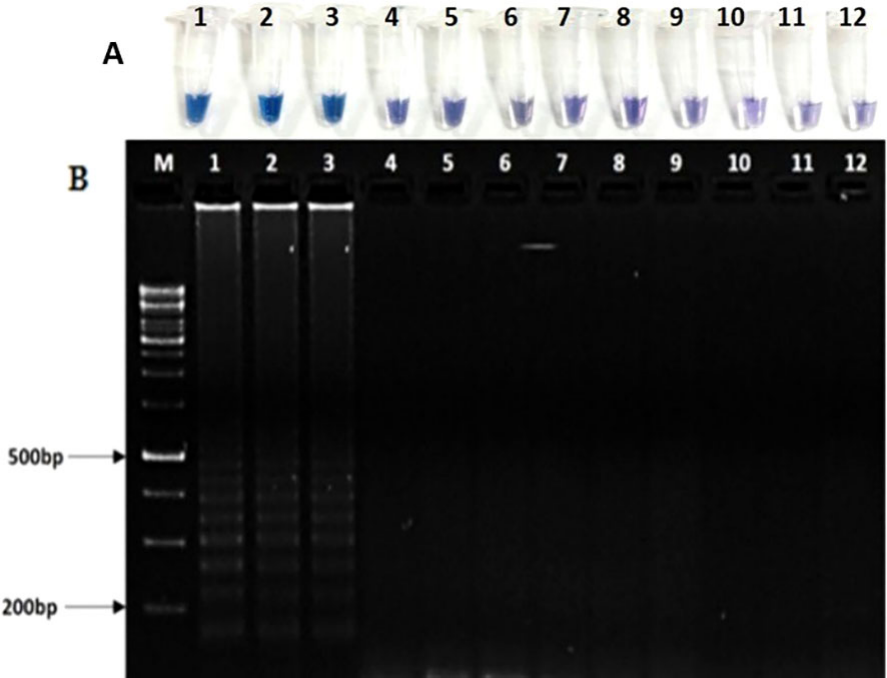
Specificity **(A, B)** of LAMP assay. **(A)** Visual detection of LAMP reaction using HNB dye. **(B)** Agarose gel electrophoresis of LAMP products. Lanes DNA template of M: 100-bp marker, lanes 1–3: *Rhizoctonia solani* isolates (TP3, TP10, TP18), 4-5: *Ustilaginoidea virens* (Uv2_4G, Uv403*)*, 6-7: *Fusarium fujikuroi* (F32, F55), 8-9: *Bipolaris oryzae* (Bo1, Bo4), 10-11: *Bipolaris sorokiniana* (Bs112, Bs69), 12: negative control.

For testing of specificity, LAMP assay was performed with template DNA of *Rhizoctonia solani* (TP3, TP10, and TP18, *Ustilaginoidea virens* (Uv2_4G and Uv 403), *Fusarium fujikuroi* (F32 and F55), *Bipolaris oryzae* (Bo1 and Bo4), and *Bipolaris sorokiniana* (Bs112 and Bs69). Under standardized conditions, amplification was found only in *R. solani* and no amplification was observed in any other sample. The same results were witnessed by HNB dye-based detection assay (**Figure 8A**) and gel electrophoresis (**Figure 8B**). The sensitivity of the LAMP detection assay was 1 ng as observed by HNB dye-based visual detection (**Supplementary Figure 2A**) and laddering pattern in agarose gel electrophoresis (**Supplementary Figure 2B**).

### Validation and application of the LAMP assay for validation of diagnostic marker in resistant and susceptible rice genotypes

**Figure 9:**
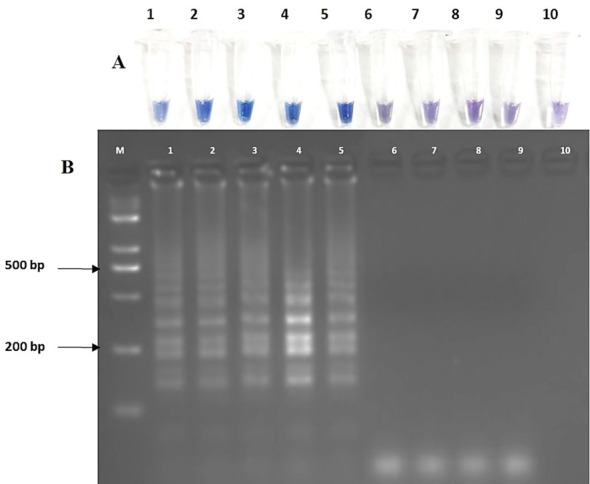
Validation and utilization of LAMP assay **(A, B)** for diagnosis of sheath blight disease of rice. **(A)** Visual detection of LAMP reaction using HNB dye. **(B)** Detection of sheath blight disease in field samples using agarose gel electrophoresis. Lanes: M: 100-bp molecular marker. Lane 1: Rasi, 2: Kalikhasa, 3: Swarna, 4: Pusa Basmati 1121, 5: Pusa Basmati 1, 6: Tetep 7: Pusa Basmati 1121 control, 8: Swarna control, 9: Pusa Basmati 1 Control, 10: negative control.

LAMP assay showed amplification of DNA in rice sheath of susceptible variety such as Pusa Basmati 1121, Rasi, Kalikhasa, Pusa Basmati 1, and Swarna. No amplification was observed in resistant and uninoculated plants (**Figure 9A**). All suspected samples taken from the field were found positive with amplification in LAMP assay developed in the study. This result was confirmed by gel electrophoresis (**Figure 9B**). LAMP-based detection assay developed in this study can be directly utilized for early and specific detection of *Rhizoctonia solani* in rice.

## Discussion

Detection and forecasting of disease as early as possible is important to manage sheath blightdisease of rice. Conventional approaches to detect *Rhizoctonia solani* are time costly and less accurate. Molecular methods to detect are rapid and accurate, and a large number of samples can be screened at a time. Therefore, in the present study, we identified a Major Facilitator Superfamily (MFS) gene unique to *R. solani*, earlier designated as AG1_BrD (Prashantha et al., 2021) based on previously available genomic information and database mining. MFS transporters have been demonstrated to be involved in multidrug resistance in fungi and are essential for the virulence and survival. In this study, we developed a unique diagnostic marker BrD3 based on a specific sequence of *R. solani* producing a fragment size of 110 bp for the detection of *Rhizoctonia solani* (AG1-IA). The specificity of the marker was tested on multiple *Rhizoctonia solani* isolates along with other rice pathogens which included *Ustilaginoidea virens*, *Bipolaris oryzae*, *B. sorokiniana*, and *Fusarium fujikuroi*. The marker was found specific to *Rhizoctonia solani* isolates, and no band was visible in other pathogens. ITS markers (White et al., 1990) were also used, which further confirmed the specificity of the marker and serves as a positive control for the pathogen DNA and reaction setup. Furthermore, we tested the marker in pathogens isolated from different hosts of the same AG group (Singh et al., 2023). Amplification was observed in all the AG1-IA isolates irrespective of host plants. Therefore, the marker developed can be utilized to detect the AG group specific to *Rhizoctonia solani*. The marker developed was observed to be highly sensitive with the detection limit of 100 pg in conventional PCR. Earlier, a PCR RFLP (Matsumoto et al., 1997) and PCR-based (Johanson et al., 1998) detection methods were developed to distinguish different *Rhizoctonia* species causing sheath blight of rice. Choudhary et al. (2020) developed a specific diagnostic marker based on the polygalacturonase (PG) gene for detection of *R. solani* AG1-IA causing sheath blight of rice. In their study, two different primers were designed, one for qPCR and another for LAMP with sensitivity up to 10 pg and 1 ng, respectively. Similarly, Lin et al. (2021) developed detection assay for sheath blight in the plant sample using TaqMan-Probe qPCR. However, they have not included soil samples for the pathogen detection. Vojvodić et al. (2019) developed conventional and real-time PCR-based specific detection markers for *R. solani* AG-2–2 causing root rot in sugar beet with sensitivity of 1 ng. Apart from this, Salamone and Okubara (2020) developed real-time PCR assay for detection of the *R. solani* AG3 group in soil. Dubey et al. (2014) developed conventional and real-time PCR-based diagnostics for *R. solani* in pulse crop. Therefore, previously different primers were designed to detect the pathogen for different techniques and sensitivity of the primers ranging from 1 to 0.1 ng, whereas the present study demonstrates a single marker (BrD3) that is applicable across conventional PCR, qPCR, and LAMP, which has been validated in plant, soil, and multiple host-derived isolates. BrD3 is a unique marker specific to *R. solani* AG1-IA, whereas most of the earlier studies used ITS and ribosomal DNA genes.

qPCR-based detection allowed quantification of pathogens accurately without being affected by the DNA of the plant. The marker developed in the present study could detect as less as 10 pg DNA concentration of *Rhizoctonia solani* in plant tissues and soil samples. Sayler and Yang (2007) developed PCR and real-time PCR assays for the AG1-IA based on the ITS region with the detection limit of 1 pg. In another study, a real-time PCR-based marker was developed for specific detection of *Rhizoctonia solani* AG-2-2 (Vojvodić et al., 2019). qPCR-based assay could detect pathogen in different genotypes, where the highest DNA concentration of pathogen was recorded in Pusa Basmati 1121, which is highly susceptible, and the lowest copy of the target gene was observed in resistant genotype Tetep, which indicates that the marker could be utilized for the resistance evaluation of rice genotypes against sheath blight disease. Su’udi et al. (2013) developed a TaqMan qPCR assay based on the *RsAROM* gene for quantification of sheath blight pathogen *R. solani* from infected rice plants, in which they quantified the pathogen DNA and was useful for evaluation of resistance. However, their study was limited to plant samples. The developed marker was further utilized for the monitoring and quantification of *Rhizoctonia solani* in soil. Maximum colonization was observed in July 2021, and the least was observed in March 2022. In conventional PCR, amplification was observed for the month of July and September whereas with qPCR, a very low pathogen load was observed in December and March samples. *Rhizoctonia solani* populations were typically lower in March compared with September due to environmental factors, particularly temperature and moisture. The pathogen thrives in warmer, wetter conditions, making late summer and early autumn (like September) more conducive to its growth and spread. Ahamad and Khan (2023) also showed the higher prevalence of the *Rhizoctonia solani* population from June to August, supporting that the population of *R. solani* was higher in the month of July in comparison with March. The marker developed in this study can detect pathogen in the plant sample and soil with a detection limit of 100 pg for conventional PCR and 10 pg for real-time PCR for both and can be utilized for detecting the rice sheath blight pathogen, quantifying fungal aggressiveness and evaluating the resistance level of rice cultivars.

The present study also reported the successful optimization and validation of a LAMP assay for the specific, sensitive, and rapid detection of *Rhizoctonia solani* in rice. The LAMP method can amplify DNA in 45 to 60 min, which is much lesser than PCR; hence, it is more rapid.

The visual detection system based on 120 μM hydroxy naphthol blue (HNB) dye provided a quick and reliable method for differentiating positive from negative reactions (Goto et al., 2009). The change in color from violet to sky blue clearly indicated the presence of amplified products. HNB is a pH-sensitive metal indicator dye that binds to magnesium ions, and color change occurs due to the consumption of dNTPs and pyrophosphate production during amplification. The use of HNB eliminates the need for post-reaction electrophoresis and allows the assay to be field deployable. In our study, LAMP specifically amplified only *R. solani* DNA and no amplification was observed in other fungal pathogens. The results were consistent in both visual detection using HNB dye and agarose gel electrophoresis, underscoring the reliability of this dual-validation approach (Tomlinson et al., 2010). The sensitivity of the LAMP assay was found to be 1 ng of genomic DNA, as evidenced by clear ladder-like banding patterns and HNB-based colorimetric assay. This level of detection is little less sensitive than conventional PCR.

Field validation of the developed LAMP assay showed successful detection of *R. solani* in leaf sheath samples from susceptible inoculated rice cultivars such as Pusa Basmati 1121, Pusa Basmati 1, Swarna, Rasi, and Kalikhasa. No amplification was observed in uninoculated or resistant plants, indicating the assay’s effectiveness in distinguishing between infected and healthy plants. Overall, the LAMP-based detection assay developed in this study demonstrates high specificity, sensitivity, and field applicability for detecting *Rhizoctonia solani* in rice. Its rapid turnaround time, simplicity, and minimal equipment requirements make it a promising diagnostic tool for early detection and disease management in rice cultivars.

The present study provides a comprehensive diagnostic platform combining conventional PCR, qPCR, and LAMP assays for the detection and quantification of *Rhizoctonia solani*. The significance of these findings lies in the ability to detect the pathogen at very low concentrations in both plant tissues and soil, which is essential for early disease forecasting and management. The developed marker opens new possibilities for precision agriculture and disease forecasting systems. By integrating this molecular diagnostic tool with field surveillance, it is possible to establish a decision-support system for sheath blight management. In future, the developed marker can be helpful for (i) pre-sowing soil diagnosis, (ii) post-harvest monitoring, (iii) in season crop monitoring, and (iv) resistance screening programs against sheath blight.

## Conclusion

The study successfully developed and validated highly specific and sensitive diagnostic tools including a novel marker BrD3 in conventional PCR, qPCR, and LAMP assay for the early detection of *Rhizoctonia solani* (AG1-IA). Its ability to detect *R. solani* in soil, plant tissues, and across different growth stages makes it a powerful tool for disease forecasting, and soil health monitoring and integrated disease management. This technique could detect levels of *R. solani* DNA in soil and help predict disease outbreaks and guide timely interventions. When combined with regular monitoring and appropriate agronomic practices, this diagnostic system has strong potential to significantly reduce sheath blight incidence and improve rice productivity.

## Data Availability

The original contributions presented in the study are included in the article/**Supplementary Material**. Further inquiries can be directed to the corresponding author.
