# Supplementary material for: Development of a multipurpose diagnostic marker using PCR, real-time PCR, and LAMP assays for rapid detection of Rhizoctonia solani in rice plants and soil
Source: Front Plant Sci. 2026 May 21;17:1724916. doi: 10.3389/fpls.2026.1724916 (PMC13233465; doi:10.3389/fpls.2026.1724916)
Supplement: Supplementary file 1 [file SupplementaryFile1.docx]

**Supplementary Table 1 -** List of isolates with accession number taken in study

| **Name of isolate** | **Pathogen** | **Place of collection** | **ITS / TEF Accession number** |
| --- | --- | --- | --- |
| TP 3 | *Rhizoctonia solani* | Laxmilunga, Tripura | ON 383492 |
| TP 10 | *Rhizoctonia solani* | Mohanpur, Tripura | ON 383508 |
| TP 18 | *Rhizoctonia solani* | Khowai, Tripua | ON 383481 |
| TP 30 Ginger | *Rhizoctonia solani* | Mohanpur, Tripura | ON383507 |
| TP 33 Turmeric | *Rhizoctonia solani* | Khowai, Tripua | ON383502 |
| TP 34 Mustard | *Rhizoctonia solani* | Khowai, Tripua | ON383491 |
| TP31 Finger Millet | *Rhizoctonia solani* | Mohanpur, Tripura | ON383516 |
| TP32 Foxtail Millet | *Rhizoctonia solani* | Laxmilunga, Tripura | ON383488 |
| TP 36 Pakchoi | *Rhizoctonia solani* | Laxmilunga, Tripura | ON383515 |
| F32 | *Fusarium fujikuroi* | Hisar, Haryana | JX 307396 |
| F55 | *Fusarium fujikuroi* | Punjab | JX307393 |
| Uv2_4G | *Ustilaginoidea virens* | Maharajganj, UP | MT312812 |
| Uv 403 | *Ustilaginoidea virens* | Cuttack, Odisha | OQ645515 |
| Bo 1 | *Bipolaris orzyae* | Ludhiana, Punjab | KU499526 |
| Bo 4 | *Bipolaris orzyae* | Haryana | KU499529 |
| Bs112 | *Bipolaris sorokiniana* | Varanasi, UP | KU 201275 |
| Bs69 | *Bipolaris sorokiniana* | Maharastra | OQ845800 |
| Bs75 | *Bipolaris sorokiniana* | Tamil Nadu | HM195259 |


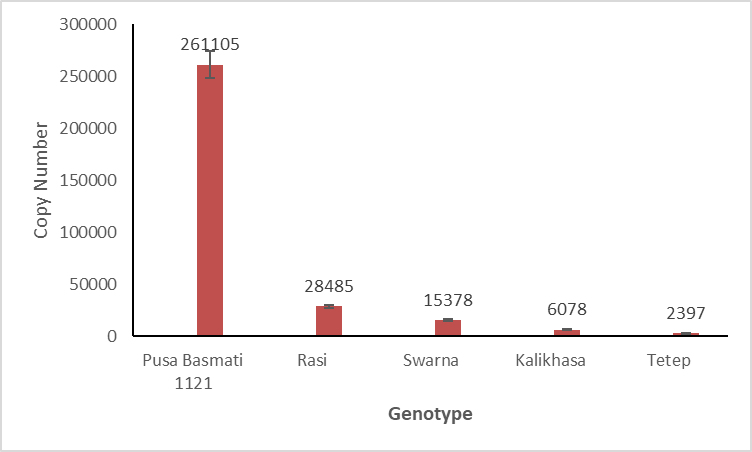


**Supplementary Fig. 1.** Detection of *Rhizoctonia solani* in different rice genotypes through copy number of target gene


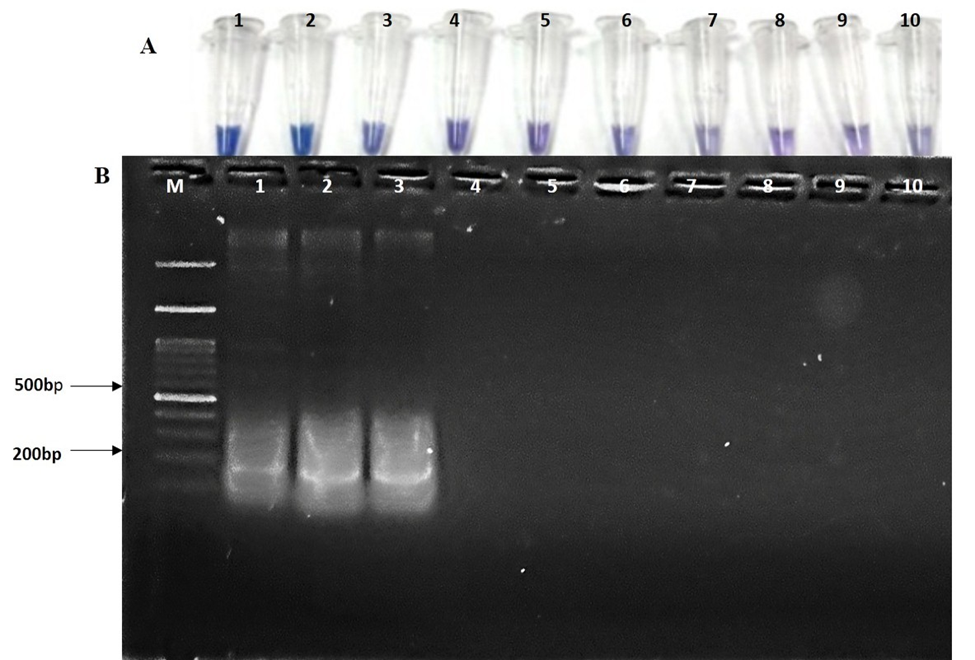


**Supplementary Fig. 2.** Sensitivity test of LAMP assay. A: Visual detection of LAMP reaction using HNB dye. B: Sensitivity test using agarose gel electrophoresis with different concentration of *R. solani*. Lanes; M (100 bp molecular marker), 1: 100 ng, 2: 10 ng, 3: 1ng, 4:1ng, 5: 100 pg, 6: 50 pg, 7: 10 pg, 8: 100 fg, 9: 10 fg, 10: sterile water
